# Estimation of consumption potentiality using VIIRS night-time light data

**DOI:** 10.1371/journal.pone.0206230

**Published:** 2018-10-26

**Authors:** Luyao Wang, Hong Fan, Yankun Wang

**Affiliations:** 1 State Key Lab for Information Engineering in Surveying, Mapping and Remote Sensing, Wuhan University, Wuhan, China; 2 Collaborative Innovation Center of Geospatial Technology, Wuhan University, Wuhan, China; 3 Research Institute for Smart Cities & Shenzhen Key Laboratory of Spatial Information Smart Sensing and Services, School of Architecture and Urban Planning, Shenzhen University, Shenzhen, China; University of Essex, UNITED KINGDOM

## Abstract

As an informative proxy measure for a range of urbanisation and socioeconomic variables, satellite-derived night-time light data have been widely used to investigate the diverse anthropogenic activities and reveal social economy development disparities from the regional to the national scale. The new-generation night-time light data have been proven to potentially improve our understanding in the development and inequality of urban social economy due to its high spatial resolution, strong timeliness and minimal background noise. These night-time light data are derived from the visible infrared imaging radiometer suite (VIIRS) instrument with day/night band located on the Suomi National Polar-orbiting Partnership (NPP) satellite. This study proposed a hybrid model to estimate urban consumption potentiality based on the comprehensive information of human activities obtained from the VIIRS night-time light data. Our method established a flexible geographically weighted regression-based estimation model based on the residential consumption data and DN values of the VIIRS data to predict the possible consumption potentiality of other urban areas in dynamic time. The experiment conducted in Guiyang, a provincial capital in China, affirms that our model is proven to have higher accuracy compared with traditional regression models and can potentially provide guidance for improved business management and increased profit.

## Introduction

Deeply involving the world development wave, the Chinese economy has experienced remarkable growth in the past 30 years, resulting in the rapid increase of their purchasing ability. The gross domestic product per capita (GDPPC) improved from 241 dollars in 1978 to 8592 dollars in 2016, with the total consumption amount reaching 5.2 trillion dollars in 2016 [1]. Although online shopping has provided an extent impact for entity retail sales, its market shares are still small compared with offline shopping. According to the National Statistics Bureau of China, the scale of online shopping was only 15% of the total Chinese retail industry in 2017. Moreover, the increasing rate of online shopping has shown a continuously decreasing trend with the gradual decline of the number of stable Internet users [2, 3]. Under the current marketing environment, consumers’ concerns about product quality and personalisation have drastically increased; thus, consumers begin to refocus on offline shopping. The proposal of ‘New Retail’, which advocates offline experience and favourable credit, enables electricity monopoly to realise the importance of offline markets and establish their offline shops, such as Jingdong, Suning and Alibaba (top e-commerce platforms in China), and even provide the same price as that in online platform to attract their offline customers.

Business managers must distinguish the spatio-temporal pattern of consumers and estimate their consumption potential to follow the trend and grasp the business chances of ‘new retail’. The appropriate estimation of urban consumption potentiality will help business enterprises maximise their expected profit. Capturing consumption potentiality has been proven important in fields, such as electronic power planning [4], food industry management [5] and urban planning [6–9]. However, precision estimation was till unsatisfactory due to the lack of high-resolution human activity information. To obtain the information of consumer potential for business management, previous studies utilised social economy statistic data, such as GDP, from Statistical Yearbooks produced in the provincial or the national scale [10]. Thus, the regional economic growth in the entire country, which provides reference for product delivery in retail business management, can be estimated. However, the huge scale of the statistical data makes the highly accurate estimation of business potential in small distinct difficult. Moreover, the static statistical data were produced in typical years. Thus, it cannot reveal the dynamic changes of social economy factors. To focus on micro scale and provide practical business strategies for small retail shops, some research considered questionnaire surveys [11, 12]. These studies considered several commercial indexes, such as income, population structure, purchasing ability and consumption habit in different regions [13].The detailed information was proven effective in providing reference for business strategies. However, the drawback of the questionnaire surveys was also evident: labour intensive and time consuming [14]. Given that the service of retail shops is consistently small, researchers begin to focus on the nearby business environment. New types of social media data, including Twitter and Sina–Weibo (a microblogging platform in China) check-in data, are introduced to investigate the spatio-temporal pattern of retail consumption with the development of mobile networks [15]. With detailed spatio-temporal human activity information, these data sources can also be combined with point of interest information to investigate the influencing factor of consumer activeness. However, the acquisition and data cleansing of the huge quantity of these social media data, which contain excessively redundant information, also require high cost. Therefore, obtaining detailed human activity information with high timeliness and low cost is profoundly important in the estimation of consumption potential for retail business management.

With the rapid development of remote sensing technology and its successful applications in solving socioeconomic problems, remote sensing data has been an important data source in social economy research. Satellite-derived night-time light data, as typical remote sensing data, are proven to have high correlation with human activities and have been widely used in investigating human activities [16] and city development over time [17]; GDP spatialization in square kilometre [18]; and other fields, including, *CO*_2_ diffusion [19], disaster distribution analysis [20] and energy consumption analysis [21]. Satellite-derived night-time light data are the electromagnetic wave information of overnight light source in visible-near infrared band through earth surface reflection. The earliest night-time light data are the stable night-time light data on the Defence Meteorological Satellite Programme/Operational Linescan System (DMSP/OLS). The DMSP/OLS night-time light data are free for public use and have coarse spatial resolution (30 arc seconds, 5 km × 5 km at nadir) and long period (1992–2013) [22]. Several limitations of the DMSP/OLS data also restrict their further application: The range of DN values in the DMSP/OLS data is small (0–63), thereby resulting in the over-saturation in city centres. Only the DMSP/OLS data from 1992–2003 are available. Thus, many studies that use the DMSP/OLS data cannot focus on social economy problems after 2013 [23]. The DMSP/OLS data are obtained by different satellites, thereby making the equality of data different even between contiguous years. In October 2011, the Suomi National Polar-orbiting Partnership (NPP) satellite with the visible infrared imaging radiometer suite (VIIRS) was launched by the National Oceanic and Atmospheric Administration (NOAA)/National Geophysical Data Centre (NGDC). As the new generation of night-time light data, the VIIRS night-time light data are superior than the DMSP/OLS data in spatial and radiometric resolution (15 arc seconds, 0.5 km × 0.5 km), radiometric detection range and on-board calibration [24]. Two types of products were available in the NPP-VIIRS website (https://ngdc.noaa.gov/eog/viirs/index.html), namely, ‘vcm’ and ‘vcmsl’. ‘vcm’ and ‘vcmsl’ are monthly averaging datasets that exclude and include the impact caused by stray light, respectively. The timeliness of the NPP-VIIRS data is better than that of the DMSP/OLS data. Moreover, the former is still updated each month, making it superior than the DMSP/OLS data in studying issues after 2013 [25].

The VIIRS data have been adopted to be a remarkable indicator of spatio-temporal human activities [26] and economics [27] owing to the aforementioned superiorities. Thus, the capability of the VIIRS data makes its use in the estimation of consumption potentiality in business management possible. Unlike other data sources, the VIIRS night-time light data have several obvious advantages for business managers. Firstly, the artificial night-time light of VIIRS can reflect the use of public and commercial lighting, which are strongly associated with the commercial activities and the state of the economy. Consequently, modelling the relationship between the VIIRS data and commercial potential for the retail industry through simple models is easy. Secondly, night-time light data are timely with low cost and flexible spatial scale. These characteristics help business managers distinguish the spatio-temporal patterns of consumers and update their business strategies, such as product distribution and advertisement strategy, each quarter or each month, in accordance to the update frequency of the VIIRS data. The VIIRS data will potentially be a new data source with great conveniences and high accuracy for retail business management.

In previous studies, a simple linear regression model was widely adopted to reveal the relationship between the VIIRS and economy data, including the estimation of the total GDP [28, 29]. The results only affirm a simple redistribution of the total GDP according to the DN values of each grid. To optimise the method, Wang [30] adopted the Huff Model in the estimation of consumption potential and site selection of new business facilities. In his study, the spatial distance between target grids and city centres was considered such that the consumption potential of a grid was not just determined by its input, but also its locations in space, which was closer to reality.

This study primarily aims to prove the potentiality of new data sources with high convenience and low cost in the estimation of consumption potential and provide other practical models for retail business management. In this study, the commercial consumption data (historical fast-moving consumer good sales data of retail shops in Guiyang, China) were combined with the VIIRS night-time light data and fed into a geographically weighted regression (GWR) model to reveal the correlational relationship between the VIIRS night-time light data and consumption potentiality. Through spatial division, our study areas were divided into thousands of small grids that contain different DN values and historical consumption amounts in 2014. These grids were used to train the GWR model, which was proven to have high accuracy in reflecting the correlation between DN values and consumption amount. After precision validation, the model was subsequently used to estimate the consumption potential from 2015 to 2017. We also compared our model with other models. The results corroborate that our model has higher accuracy than traditional regression models and is then used to estimate the consumption potential from 2015 to 2017. The research can potentially provide guidance for business managers to formulate improved retail business strategies.

The remaining contexts are organised as follows: Section 2 introduces the study area and data source used in the research. Section 3 introduces the methods, including the establishment of the GWR-based regression model, the spatial inequality index and the potential estimation process. Section 4 conducts the experiments in Guiyang, China, and regions in Guiyang were distinguished according to their market values. Section 5 provides the conclusions and directions for future works.

## Study areas and data source

### Study areas

**Fig 1:**
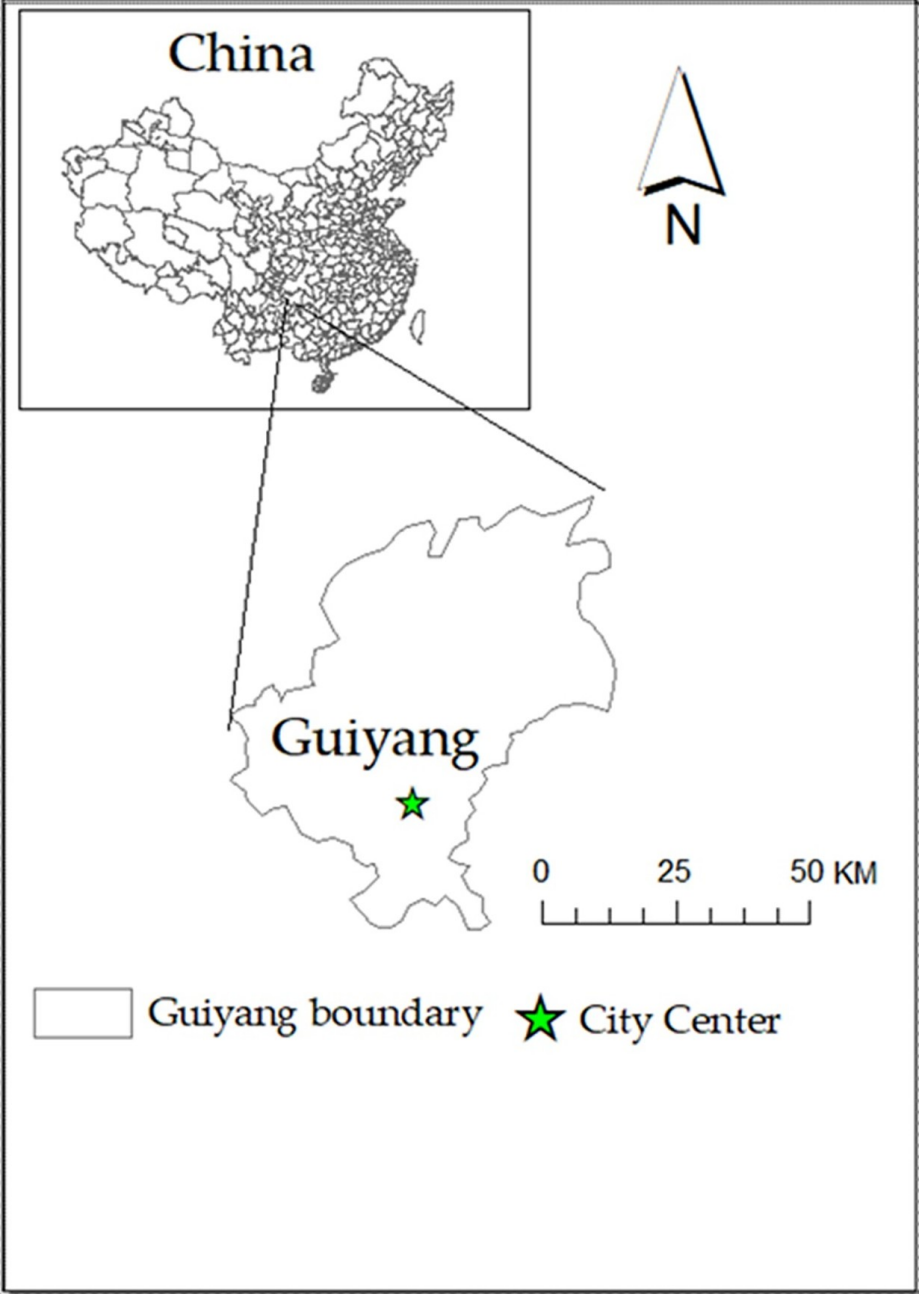
Location and boundary of Guiyang.

Located in the South Central of China, Guiyang is the capital city of Guizhou Province. Fig 1 shows the location of Guiyang. As one of the biggest central cities in Southwest China, Guiyang is also the national transportation hub and is a famous tourism city in China. The natural scenery in Guiyang is unique due to its moist climate because of high average elevation (more than 1100 metres. Known as the “Forest City‘, the natural scenery in Guiyang, such as the famous Huangguoshu Mountains and Waterfalls, attracts tourists from all over the world. The overall territory area of Guiyang is 8034 square kilometres, which includes six regions and three county-level cities. The permanent resident population in Guiyang is over 4.5 million in 2016.

The ‘One Belt, One Road‘ programme, which is an initiative proposed by China in 2013, aims to establish a huge economic cooperation platform with distinction from China to Europe[31]. The social economy has experienced prominent development, with the total GDP reaching 56.35 billion dollars and GDPPC reaching 10.79 thousand dollars, which is more than the average level of China. The big data industry in Guiyang has been developing rapidly, and the national big data-centre has been set in Guiyang, making it become the gathering place for the information industry and data trading businesses. Guiyang is also a pilot city of the circular economy in China. Thus, many new economy patterns have been developed in Guiyang. The rapid economic development has attracted many foreign investments to establish their business chains in Guiyang, thereby making the estimation of consumption potentiality in different areas and the distribution of the commercial resource in reasonable ways challenging for business managers.

### Data source

#### VIIRS night-time light dataset

The night-time light data used in this study were the monthly VIIRS night-time light data that range from 2014 to 2016. The data were obtained from the official website of NOAA’s National Centres for Environmental Information (https://ngdc.noaa.gov/eog/viirs/index.html). Three types of dataset were provided in the website: Raw (RDR), sensor (SDR) and environment data records (EDR) [32]. SDR is the production that contains radiance. Thus, SDR is used in this study. The SDR data are open access for the public and updated each month from May, 2011 to April, 2018. In the original dataset obtained from the website, the effects of aurora, fires, boats and other ephemeral lights have not been removed. Generally, the first step is to remove the effects of these background noises. In this study, the following preparations were taken before the analysis of VIIRS. (1) For the reduction of the influence of background noise and abnormal lights, the maximum radiance value derived from the city centre was set as the upper threshold. We distinguished two city centres that aggregated many pixels with high DN values. Moreover, the maximum DN value in the two centres was 125.8. Therefore, the DN value of −125.8 was set as the upper threshold. Pixels with large DN values were considered the outliers and should be set as 0. (2) The low threshold was obtained from the non-residential areas. The process was realised through the overlay of the night-time light image with land use data extracted from the Landsat8 dataset. An average DN value of −4.7 of pixels, containing lakes and rivers, was set as the lower threshold. In this way, the light values that were smaller than the lower threshold or larger than the upper threshold were treated as noises, and their values were set as 0.

**Fig 2:**
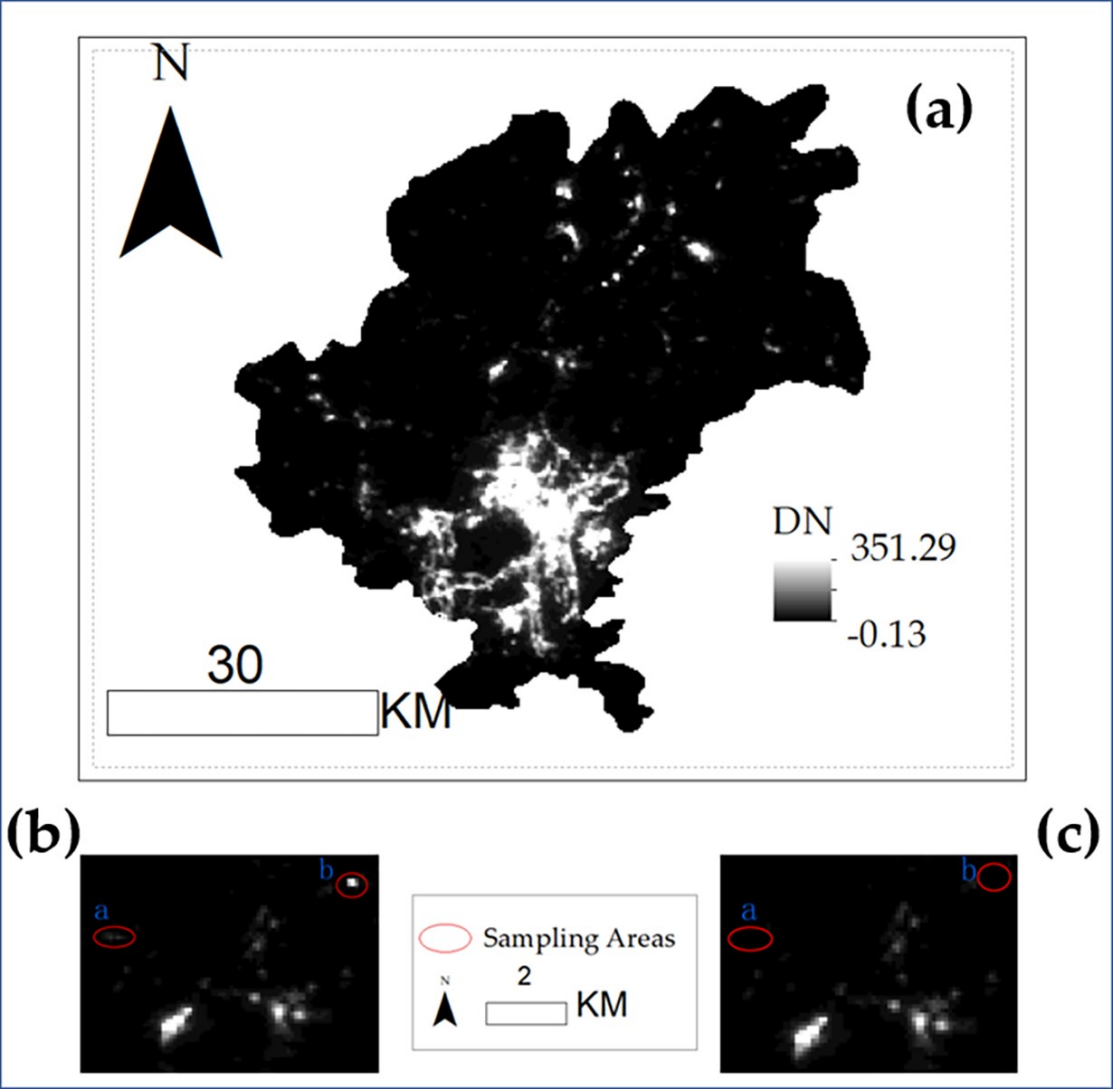
Original data set and examples of data preprocessing. (a) the original night-time light data in Guiyang during September, 2016; (b) the original data with abnormal values; (c) the data after data preprocessing and the abnormal points in a and b were eliminated.

The night-time light dataset in 2016 was selected as an example to show the correction process in our study. Fig 2(A) shows the original image in the 2016 dataset, which has been clipped by the mask of Guiyang City. The maximum digital number (*DN*) value in the original image was 351.29, which was far more than the regular *DN* values of residential areas, because the monthly night-time light data contain unstable or instantaneous light information. We found that the maximum *DN* value was 125.8 and that 248 pixels with larger *DN* values were mainly distributed far away from the city centres through the sampling of the pixels in the city centre. The *DN* values of these regions, which were far away from the city centres, were distinguished as abnormal values and adjusted to the background values of 0, such as region *a* (Fig 2(B) and 2(C)). For regions with large *DN* values but located in the urban areas, the *DN* values were adjusted to 125.8. Another influence factor was the background noises that exist in the original image, which could result in negative values or missing data in some pixels, such as region *b* (Fig 2). The *DN* values of these regions were also set as 0 to eliminate the errors.

The *DN* value of each pixel was rounded down to integer because the *DN* values of night-time light data were floating-point numbers, which could not be analysed in ArcGIS10.3:

$${DN}_{new}=\lfloor DN\rfloor .$$ (1)

**Fig 3:**
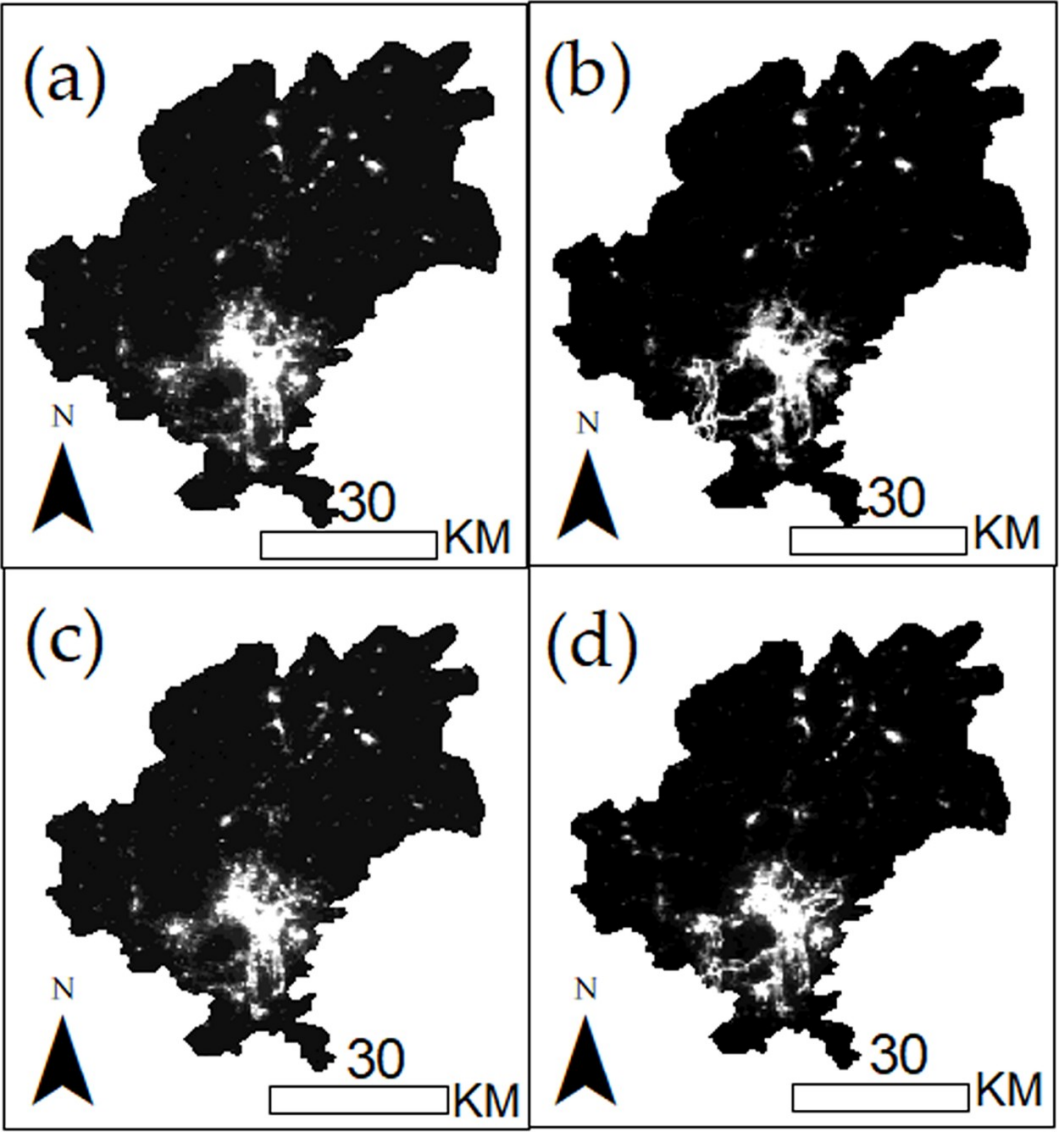
Images from 2014 to 2017 after correlation.

Images after processing are shown in Fig 3, and the *DN* values of which have been rounded down to integers.

#### Consumption data in Guiyang

The retail sale data in Guiyang were obtained, including the location information and monthly fast-moving consumer goods (FMCG) (i.e., clothes, food and drinks) sales of 5837 retail shops of Guiyang between January,2015 and December,2015. These shops include supermarkets, chain stores and groceries. The retail data were provided by local companies for research purposes (https://github.com/SuperHandsomBoy/Sales-data2.git). Table 1 shows the samples.

**Table 1:** Examples of consumption data.

Retail ID	Type	Name	Lon	Lat	Sales (US dollars/month)
54116864	Small supermarket	Hualian Supermarket	106.717	26.605	68,152
54116360	Small supermarket	Yonghui Supermarket	106.715	26.583	41,516
54127706	Chain convenience store	Hanlejia 24h chain store	106.719	26.571	18,196
54130581	Groceries	Youjia Grocery	106.695	26.335	7289

The number of retail shops in each Guiyang distinct was counted through spatial statistics and is shown in Table 2. The table shows that retails in Nanming distinct. possessed the highest sales performance, with the average sales of 1386 retails, reaching 1150 dollars/month. The left areas have three distinct, namely, Yunyan, Huaxi and Guanshanhu, and their average retail sales are more than 1000, corresponding to 1016, 1076 and 1107 dollars/month, respectively. These regions were the core areas in the downtown of Guiyang. The three distincts with average sales of less than 800 dollars/month were Qingzhen (748 dollars/month), Baiyun (750 dollars/month) and Xifeng (723 dollars/month).

**Table 2:** Information of retail shops in each distinct.

ID	Distinct	Shop number	Average sales (dollars/month)
1	Yunyan	1851	1016
2	Nanming	1386	1150
3	Huaxi	713	1076
4	Qingzhen	327	748
5	Kaiyang	186	796
6	Baiyun	353	750
7	Guanshanhu	418	1107
8	Xiuwen	153	860
9	Xifeng	204	723
10	Wudang	246	927

Table 2 shows that great imbalance existed amongst these regions. The sales performance of the core areas of Guiyang are more obvious than that of suburban areas. The retail shops we obtained were not all the shops in Guiyang. However, they were all typical retail shops in each distinct with high activeness. We have conducted some pre-processing steps, such as removing inactive shops and selecting the best time period, to enable the data to reflect the actual market situation.

## Method

### GWR model

In previous studies about night-time light data, the ordinary least square models (OLS) were often adopted to model the relationship between independent and dependent variables, such as the study of *CO*_2_ diffusion [19] and GDP spatialization [18]. In fact, the OLS models were only the simplified description of these relationships, which impertinently suppose the spatial equality of social economy factors. However, the correlation between the DN values and the consumption potential cannot be modelled only through a set of constant parameters. However, according to the ‘second law of geography’, spatial heterogeneity widely existed even in any social contexts [33], indicating that the pixel locations also affect their prediction values. This basic rule is ignored, and mistakes due to low accuracy often occurred in the results of OLS when adopted in the city space. In this study, the geographical weighted regression (GWR) model was introduced to model the relationship between *DN* values and market sales performance. The GWR model is an improvement of traditional OLS models, in which spatial locations are considered in the model [34]. The GWR model is better in describing DN values and consumption potential than OLS models. The OLS model, which imitates its ability in reflecting complex spatial issues, only has one set of parameters. Huge differences between cities and different parts of cities, such as urban and rural areas, exist due to different development levels. The same DN values in urban and rural areas may represent different potentials. Therefore, the pixel locations should be considered when predicting their consumption potential. The parameters of the GWR were set according to its DN values and locations, thereby making the model closer to reality. In this study, the GWR model was established:

$$y_{i}=\beta_{0}(u_{i},v_{i})+\sum_{k=1}^{p}\beta_{k}(u_{i},v_{i})x_{i}+\varepsilon_{i}\;,i\in [1,n],$$ (2)

where (*u*_*i*_,*v*_*i*_) represents the spatial locations in point *i*, *β*_0_(*u*_*i*_,*v*_*i*_) represents the constant term, *β*_*k*_(*u*_*i*_,*v*_*i*_) represents the regression coefficient (the spatial weight function), *x*_*i*_ represents the value of independent variable in point *i*, *ε*_*i*_ represents the error term and *y*_*i*_ represents the dependent variable in point *i*. The regression coefficient *β*_*k*_(*u*_*i*_,*v*_*i*_) is identical to the weighted least squares regression, where the weights are computed on the basis of the Euclidean distance between objects. The regression coefficient is described in [35]:

$$\beta_{0}(u_{i},v_{i})={\left(X^{T}W(u_{i},v_{i})X\right)}^{-1}X^{T}W(u_{i},v_{i})Y,$$ (3)

where *X* represents the matrix formed by independent variables (*x*_*i*_) and *Y* represents the vector formed by dependent variables (*y*_*i*_). Moreover, the most important parameter *W*(*u*_*i*_,*v*_*i*_) represent a weight matrix, which is determined by the spatial Euclidean distance between objects. The weight of a point will show an inverse relation with its distance to other objects, thereby ensuring that the objects near point *i* have more influence on the results than those farther away. In GWR, the confirmation of the spatial weight matrix is of great importance for the accuracy of the model and should be conducted before the analysis. The spatial weight function was often determined by kernel functions, including Gaussian, bi-square and tri-cube types [35]. In this study, the commonly used Gaussian function was adopted:

$$W_{ij}=ex\;p\;\left(-\frac{d_{ij}^{2}}{b^{2}}\right),$$ (4)

where *d*_*ij*_ denotes the Euclidean distance between *i* and *j*; and *b* denotes the bandwidth, which is used to describe the attenuation of spatial weights. Thus, the spatial weight matrix can be finally formed. Furthermore, each value in the matrix described the proportion of the point’s distance to nearby objects in the entire distance records.

### Spatial division and estimation of consumption potentiality

Investigating the consumption potential in regional scale is appropriate considering that the service area of retail shops is often regional. Therefore, spatial division, which will divide the whole study area into numerous geographical units, is conducted before the analysis. Three methods for space division are commonly available, including spatially weighted Voronoi diagrams, Delaunay triangulation and grid diagrams [30, 36]. Voronoi diagrams and Delaunay triangulation consider the actual values of input and divide space into irregular units based on their values. The units should be nonoverlapping. The problem is when objects are nearby each other, the units that formed will be very small, even when they have high values. Moreover, the units formed by isolated points will be very large even when their actual values are very low. Therefore, the two methods were not appropriate for investigating retail shops, which often have relatively constant service areas of approximately 500 *m* × 500 *m*[37]. The grid diagrams are more appropriate than other methods because of their continuity in spatial division. The grid-based method divides the study area into continuous grids with constant size. Therefore, we finally selected 500 *m* × 500 *m* as the spatial division size, which is also close to the spatial resolution of the VIIRS night-time light data (400 *m*~800 *m*).

The specific spatial division was conducted as follows:

1. The VIIRS night-time light data that cover the entire Guiyang City was resampled into the uniform 500 m spatial resolution.
2. The resampled image was converted into a 500 *m* × 500 *m* vector diagram. Each vector grid in the vector diagram was coincided with a pixel in the resampled image and contained the grey value.
3. The retail shops, which were represented by vector points, were overplayed into the 500 *m* × 500 *m* vector diagram. The number and sales of retail shops that fall into each vector grid were summed and recorded.

The grids after overlaid analysis were set as the training and test data set of GWR regression model, where the DN values in each grid were set as the input of the model and total sales were set as the output of the model. In the process, two steps were considered to improve the processing precision. (1) When nearby the distinct boundary, the cells were segmented by the administrative boundary, and *DN* or sales vales were set according to the size of the acquired area in each distinct. (2) In a few cells, the *DN* values were 0, but their sales were more than 0. These places were believed to be temporary construction sites or clearance areas, which did not have stable market values. Thus, these cells were removed from the dataset. After the process, 1227 cells were left and selected as the study data set.

### Distinguishing grids with the highest consumption potential considering regional development differences

High consumption potential is definitely an important index for business management. However, it should not be the only index for business management. For instance, the consumption potential of capital cities is higher than that of nearby county-level cities. The consumption level of city centres is also higher than that of other regions even in the same city. However, only opening retails in city centres and ignoring the market in downtown or small cities, where competitions and rent cost are often low, are unreasonable. Through the estimation of the consumption potential of Guiyang, regions with high potential will distributed intensively in the two core areas, namely, Yunyan and Nanming. To explore the market values of each area more reasonably, we proposed an evaluation index based on the consumption potential of grid and development speed of its belonging distinct. We defined variable $V_{ik}^{\prime }$ to describe the market values in each region:

$$V_{ik}=\left\{\begin{array}{c} P_{ik}\times I_{k}\;,P_{ik}>1000(dollars/month)\\ 0,P_{ik}\leq 1000(dollars/month) \end{array}\right\}\;,k=1,2,3\ldots ,10,$$ (5)

$$I_{k}=Average\;\left(\sum_{y=2014}^{y=2016}\frac{{Theil}_{k}^{y+1}}{{Theil}_{k}^{y}}\right),$$ (6)

$$V_{ik}^{\prime }=Normalization(V_{ik}),$$ (7)

where *V*_*ik*_ is a comprehensive consideration of the consumption potential and development level of its belonging distinct. *P*_*ik*_ represents the estimated consumption potentiality in grid cell *i*, located in distinct *k*, and *I*_*k*_ represents the average development speed of distinct *k*. ${Theil}_{k}^{y}$ represents the Theil index in distinct *k* in time *y*. The Theil index is used to describe the different developing levels amongst regions and was proposed by Theil in 1967 [38]. In this study, the Theil index is defined as follows:

$$Theil=\sum_{i=1}^{N}\left({DN}_{i}/{DN}_{sum}\right)\times \ln\left(\frac{\frac{{DN}_{i}}{{DN}_{sum}}}{\frac{S_{i}}{S_{sum}}}\right),$$ (8)

where *DN*_*i*_ denotes the *DN* value in region *i* and *DN*_*sum*_ denotes the total *DN* values in all the regions of Guiyang City. *S*_*i*_ denotes the sales data in region *I*; *S*_*sum*_ denotes the total sales data of all regions in Guiyang City. For the elimination of the inconvenience caused by large units in *V*_*ik*_, the *normalization* process was conducted to obtain the new index ($V_{ik}^{\prime }$) between 0 and 1. Moreover, cells with larger $V_{ik}^{\prime }$ values possess high market values. Through the process, 1227 cells were left for the analysis, and the $V_{ik}^{\prime }$ values of which were larger than 0.

## Experiment results

### Establish of the GWR model and estimation of consumption potentiality

The relationship between night-time light data and the actual consumption potentiality was firstly established through the historical sales information and DN values in the grids of Guiyang City in 2015.The fivefold model was adopted to train the model and evaluate its accuracy. A total of 1227 grid cells prepared through spatial division were roughly divided into five parts (i.e. part 1; part 2, which contains 246 grids; and parts 3–5, which contain 245 grids because 1227 cannot be divided exactly by 5), with each containing 20% of the whole grids. In each time, one part will be selected as the test sample, and the four remaining parts are selected as the training sample. The accuracy of each time was calculated (Table 3).

**Table 3:** Accuracy of training with different data sets.

Time	Training sample	Test sample	R2
1	Parts 1, 2, 3 and 4	Part5	0.898
2	Parts 1,2, 3 and 5	Part4	0.835
3	Parts 2, 3, 4 and 5	Part1	0.794
4	Parts 1, 2, 4 and 5	Part3	0.912
5	Parts 1, 3, 4 and 5	Part2	0.769
**Average**			0.841

The average accuracy is 0.841, showing that the model we established could be appropriate for describing the relationship between night-time light data with consumption data. The results also confirmed that, when part 3 was set as the test data, the GWR model obtained the highest accuracy (0.912). Therefore, training sample Parts 1, 2, 4 and 5 could reflect the major characteristics of the entire dataset. Moreover, the GWR model trained through the four parts were subsequently confirmed as the final estimation model.

After establishing the GWR models, it was applied to the dataset of three years, that is, 2014, 2016 and 2017. The sales performance of the six distinct in 2014–2017 was then estimated accordingly, and the results were shown in Table 4.

**Table 4:** Estimation results of consumption potentiality in each year through GWR.

Time	Consumption potentiality (billion dollars/year)	Increasing rate
2014	1.19	/
2015	1.29	8.4%
2016	1.40	8.5%
2017	1.57	12.1%

**Fig 4:**
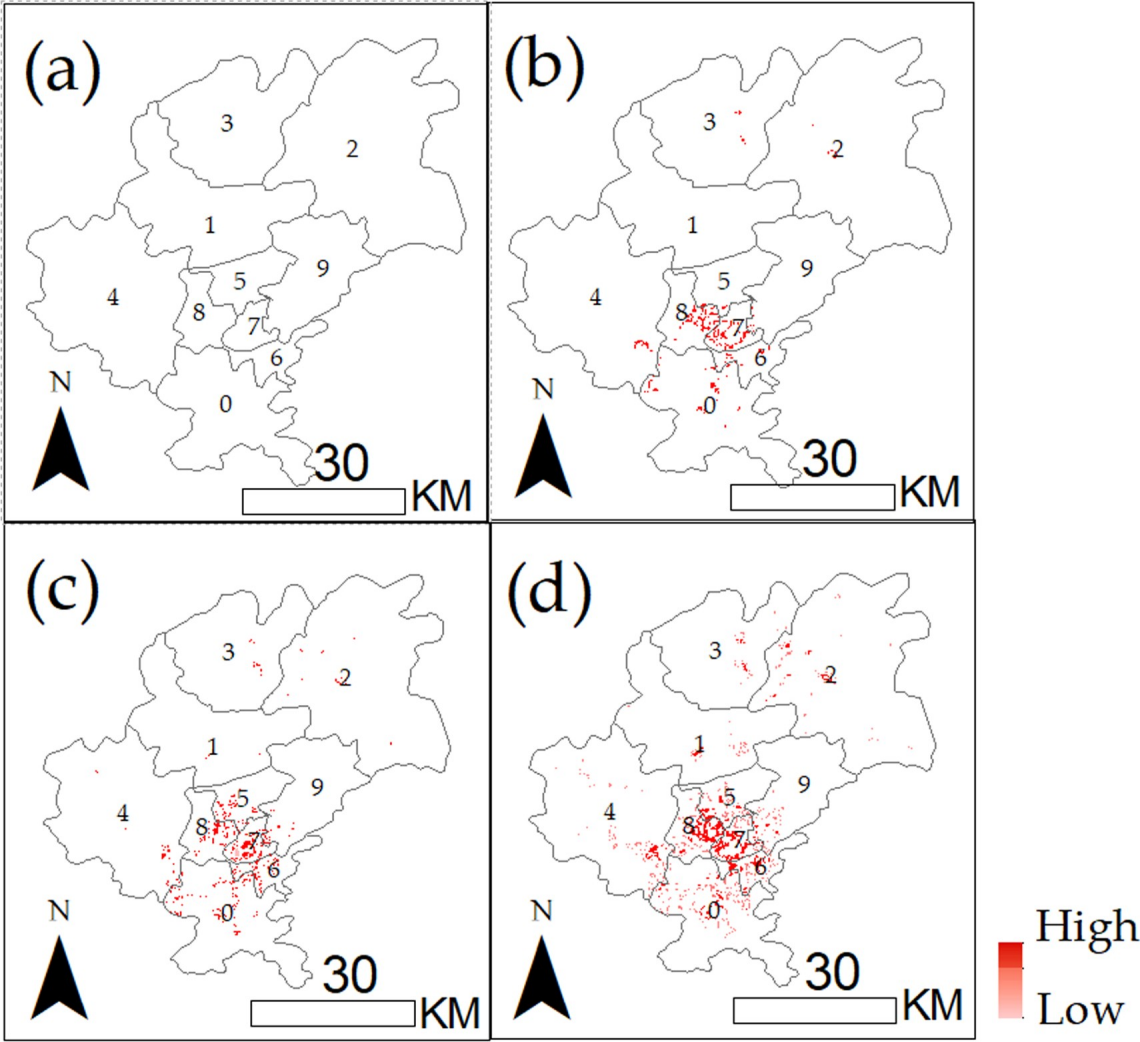
Growth of consumption potentiality from 2014 to 2017. The red colours with different degrees were used to show the rangeability of consumption potentiality.

Table 4 indicates that the consumption potentiality of Guiyang gradually increased from 2014 to 2017, from 1.19 billion dollars in 2014 to 1.57 billion dollars in 2017. Moreover, the increasing rate was stable at 8.4% and 8.5% from 2014 to 2016. The consumption potentiality increased remarkably in 2017, with the promotion over 0.17 billion dollars and increasing rate reaching 12.1%. For the determination of the growth in space, the consumption potentiality in micro-scale was estimated. Fig 4 shows the increase of consumption potentiality in different areas.

Fig 4 shows the development of sales performance in Guiyang City from 2014 to 2017. The sales performance in 2014 was set as the reference, and the degree of red colour shows the increasing ratio compared with that of last year. The results in Fig 4 affirm that the total increase of consumption potentiality was relatively stable between 2014 and 2016 and that great differences existed in each distinct. Guiyang experienced a relatively high increase in the east part of the distinct during 2016 and 2017. The most evident increase of consumption potentiality was in the south regions, including Guanshanhu, Huaxi and Nanming, which were the core areas of Guiyang. In nearby counties, Xiuwen, Wudang and Qingzhen have obtained a great potential increase from 2015 to 2017.

### Comparison of the performance of different models

The performance of the model was calculated after the estimation processes. We compared the performance of our model and several other methods that are frequently used in estimation and prediction tasks. The akaike information criterion (AIC) index, which is the comprehensive consideration of residual and model complexity, was used to evaluate the performance of the models. Small index indicates that the model’s performance is remarkable. AIC can be calculated as follows:

AIC=2K−2ln(L), (9)

where *K* denotes the number of parameters of the model and *L* denotes the likelihood function of the model. The five parts were divided and used for comparison. In each time, one part will be selected as the test sample, and the four remaining parts are selected as the training sample. Moreover, the average AIC will be set as the final AIC index of the model. Table 5 depicts the results.

**Table 5:** Comparison amongst different methods.

Model	AIC
Linear Regression	617.138
Polynomial Regression	484.152
OLS (least square regression)	504.742
GWR-based model (Ours)	255.286

Table 5 shows that the AIC of linear regression model was 617.138. *R*^2^ of the polynomial regression model and that OLS values were 484.152 and 504.742, which were smaller than that of the linear regression model, respectively. This phenomenon indicated that the simple linear regression model could not effectively reveal the rules in actual social economy factors. The AIC of the GWR-based method is 255.286, which is much smaller than that of the three other methods. Therefore, the performance of the GWR-based model was better than that of the three other methods. Thus, the GWR-based model remarkably reflected the consumption potentiality with the VIIRS data.

### Distinguishing high consumption potential areas in ten distinct

For the estimation of the market values in micro-scale, $V_{ik}^{\prime }$ of each grid cell was calculated, and the results were counted in Table 6. Table 6 shows that three classes were distinguished according to their *V’* values: Classes 1, 2 and 3. Class 1 refers to places with high *V’* values (0.75–1). The estimation of consumption potentiality in these places increases rapidly year by year, indicating that the development speed in these places was higher than the average level. Thus, economic vitality in the regions was stimulated, and their consumption potentiality increases continuously. Class 1 refers to places with moderate *V’* values (0.35–0.75). The increasing contribution rate in these places was stable, indicating that the commercial values in these places were sustainable without much risk. The consumption potentiality in these places increases more slowly compared with that of Class 1. Class 3 refers to places with low *V’* values (0–0.35.) These regions were mainly distinct of old neighbourhoods in the city, the consumption potentiality in these regions was stable, and their increasing speed declined due to the limitation of urban size. The regions were mainly mature economy areas, and the consumption potentiality in these regions has reached their maximum value. Thus, they could not ensure increasing profits for retail shops.

**Table 6:** Number and *V’* of each class of sites.

Classes	Number	*V’*
Class 1	293	0.75–1
Class 2	347	0.35–0.75
Class 3	587	0–0.35

**Fig 5:**
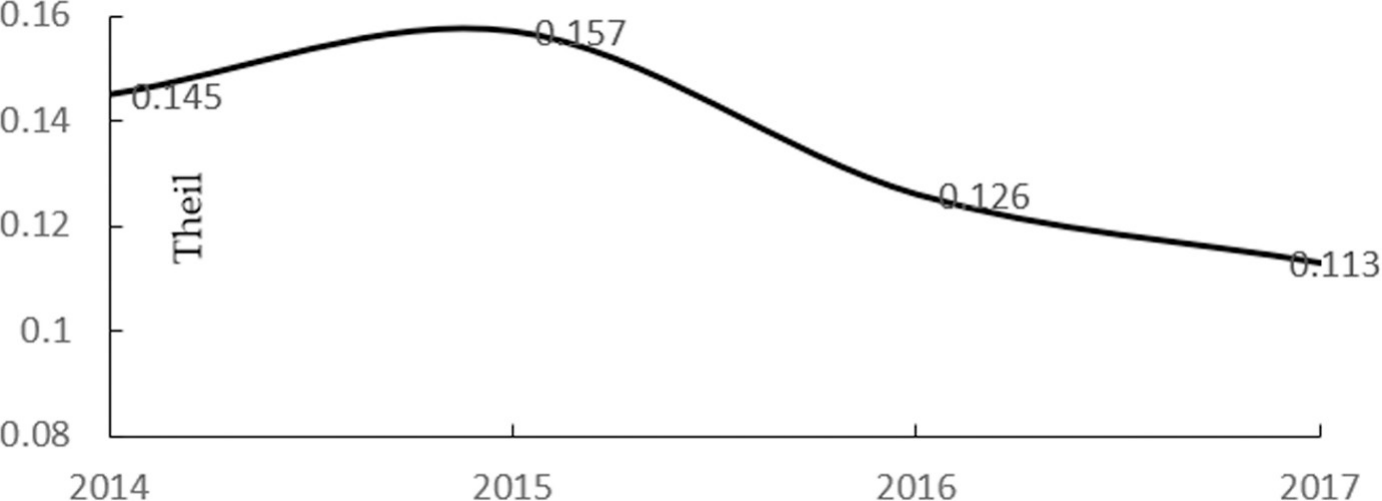
Market values in different sites. **Grids with red colours were regions with high market values.** Grids with orange colours were regions with moderate market values and grids with transparent colours were regions with low market values.

The volume of consumption potentiality and the high increasing rate of consumption potentiality are both important indexes for business managers. Although the 73 sites in Class 1 all possessed the highest market values with their *V’* values larger than 0.75, the actual consumption potentiality still shows difference. Some business managers may mainly focus on the increasing rate and ignore their current sales performance. Regions with high development space were often suburbs or new urban districts with low rent and visitor flow but occupied with significant increasing rates. Other business managers may focus on the stability and low risk of commercial places and ignore the high rent cost in these places. In this way, the places with high market performance but relatively low increasing rate will be a remarkable choice for them. As the *V’* values were the combination consideration of market quantity and increasing rate, business managers can select appropriate sites according to their preference. In this study, the places of Class 1 were distinguished into three types with different colours as an example, as shown in Fig 5, where the places in Guiyang were divided into three colours based on their comprehensive performance. The number of each type of places was counted and shown in Table 7.

**Table 7:** Number and consumption potentiality of each site.

Classes	Number	Colour	Potential (dollars/month)	I
Sites 1	64	Red	5000–7000	>1.065
Sites 2	117	Orange	7000–10000	1~1.065
Sites 3	112	Transparent	>10000	≤1

Fig 5 and Table 7 show 64 regions with red colour were places that possessed consumption potentiality 5000–7000 dollars/month and high increasing rates (I>1.065). The regions would be recommended to the investors because of their high market prospect although their temporary consumption potentiality was not very high. It will be a good choice for business managers to locate their retail chains or supermarkets in these regions. A total of 117 regions with orange colour refer to places that possessed consumption potentiality of approximately 7000–10000 dollars/month and moderately increasing rates. In these places, the potential was higher than those in red regions, but their increasing rate was slower (1<I≤1.065). These places were mainly distributed nearby the city centres and could be an eclectic choice for business managers. A total of 112 regions with a transparent colour refer to places that possessed consumption potentiality more than 10000 dollars/month and low increasing rates (0≤I≤1). These places were mature business centres and were suitable for business managers who wanted to avoid risks and obtain stable profit. The biggest challenge for retails in these places was to maintain their current situation and avoid cutthroat competition with nearby places. The distinction of these regions could help business managers discover places with high market values and formulate the most fitting business strategies to realise their expected profit.

### Exploration of the economic development tendency

**Fig 6:**
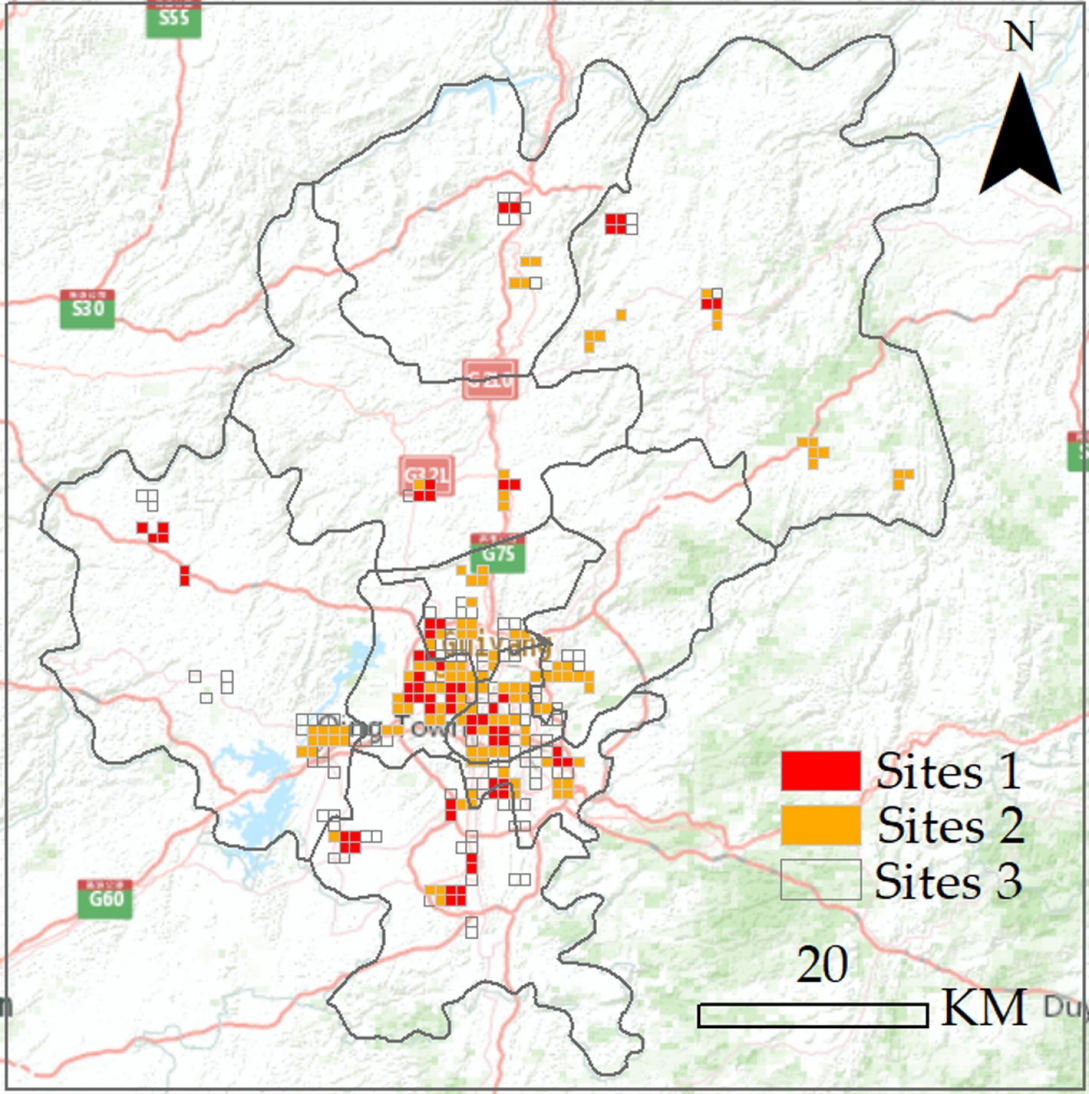
Changing tendency of consumption potential of each distinct between 2014 and 2017.

For the revelation of the detailed changing tendency of consumption potential in each region, the consumption potential of each region and county was calculated during 2014–2017 and is shown in Fig 6.

Fig 6 shows that the regional difference of the social economy development is high. The overall contribution rate was contributed mainly by the core regions of Guiyang, including Yunyan, Nanming, Baiyun and Huaxi. Three types of regions were distinguished according to their contribution rate: the increasing regions, including Xifeng, Kaiyang and Guanshanhu; the stable regions, including Qingzhen, Huaxi, Naming and Wudang; and the decreasing regions, including Baiyun and Yunyan. Amongst these regions of Guiyang, Guanshanhu has gained a remarkable increase of consumption potentiality, which can be reflected by its increasing contribution rate to the Theil index. The contribution rate of Guanshanhu increased from 8.7% to 10.6% during 2014–2015 and from 10.6% to 12% and 12.8 during 2015–2016 and 2016–2017, respectively, which show the huge benefits of setting up the big data centre in Guanshanhu. The contribution rate of Guanshanhu even became the highest in 2017. Fig 6 also shows the continuous development of Xifeng and Kaiyang with the contribution rate increased from 5.5 to 7.1 and 7.4 to 9.7 during 2014–2017. The influence of the core regions of Guiyang to the overall index decreased during the 4 years, showing that the economic growth speed has reduced with the industrial transformation in these years. Baiyun distinct acquired the highest decrease, with the contribution rate decreasing from the highest rate of 17.9 in 2015 to the lowest rate of 11.7 in 2017. The increasing rate of consumption level in Huaxi and Nanming was stable and positive (Fig 6). The total results affirmed that the economic center has been moving from Baiyun, that is, to Guanshanhu distinct in the core cities. Moreover, the development balance amongst these regions, particularly in core cities and nearby counties, has been improved, which would bring positive effects to the equal resource allocation and resident income.

## Discussion

Benefiting from the development of remote sensing technology, night-time light data, as one of the new typical remote sensing data, has been widely used in the study of social economy. The VIIRS night-time light data, with their high revolution and availability for the public, will be a great potential in the study of other microcosmic and complex socioeconomic issues. Combining the business sales data with the VIIRS data, this study firstly proved the potentiality of the VIIRS data in the reflection of regional consumption potential and its ability to predict future business performances.

However, some limitations still exist in the current analysis. The first limitation is that the original VIIRS data generally cover non-residential areas, such as parks, mountains and lacks. The DN values in these regions have negative influence on the estimation accuracy. Although the threshold value method has been used in this study to weaken the influence, large room for improvement to cities with much more lacks or parks, such as Wuhan and Hangzhou, still exists. Therefore, some external data source, including Landsat and OpenStreetMap data, can be introduced to help distinguish these non-residential regions and eliminate their negative influence. The second limitation is that we just take analysis of the yearly VIIRS data and yearly sales data. Actually, the VIIRS data are updated per month, making it possible to reveal the monthly consumption potential changing and help business managers conduct more timely strategies. However, the sales data of some retail shops were provided per quarter or per year. The monthly sales data in these shops are limited. In future works, more detailed business data can be obtained through business companies or social media platforms, thereby providing more potentiality in predicting consumption potential and optimising business strategies.

## Conclusion

With the rapid development of remote sensing technology, the VIIRS satellite-derived night-time light data were proven to have high correlation with human activities and have been used in improving business management [39]. In this study, the satellite-derived night-time light data were firstly used in the estimation of consumption potentiality in China. As a typical commercial data source, the consumption potentiality is closely related to the human settlements and human activities. It can be used to reflect the urban development such that the VIIRS night-time light data can be a new choice for the research of people’s consumption potential. However, previous studies often evaluated the social economy factors based on simple linear model without considering the development degree of different locations. For the improvement of the estimation accuracy, a hybrid model was established through the GWR method and was proven superior than the three other regression models used in previous studies. Through the experiment, three classes of places were distinguished according to their consumption potentiality. The main contribution of this study is concluded as follows:

1. The study introduced a new data source for business management. Compared with traditional statistical data, the VIIRS night-time light dataset is easy to acquire and shows high correlation with human activities. These characteristics drastically provide huge convenience for the timely estimation of market demand, thereby improving profits for the business management.
2. In this study, a GWR model was established for more accurate estimation of consumption potential. The model could set its correlation coefficient by considering the development degree of each region, making it capable to solve business problems in different cities.

In the context, the historical consumption data just covered the year 2015, and the changes of sales performance over time were not considered. Moreover, the distribution of consumption data was not homogeneous, which may influence the accuracy in establishing the model. In the future work, we will consider the temporal changes of consumption data and the usage of a more external dataset to obtain accurate and precise results.
